# Supplementary material for: Bayesian refinement of protein structures and ensembles against SAXS data using molecular dynamics
Source: PLoS Comput Biol. 2017 Oct 18;13(10):e1005800. doi: 10.1371/journal.pcbi.1005800 (PMC5662244; doi:10.1371/journal.pcbi.1005800)
Supplement: S1 Archive — (BZ2) [file pcbi.1005800.s011.tar.bz2 › gromacs_4.62_bayesian_saxs_refi/share/html/online/g_wheel.html]

g\_wheel


| g\_wheel Main Table of Contents |

**VERSION 4.6  
Sat 19 Jan 2013**

---

### Description

g\_wheel plots a helical wheel representation of your sequence.
The input sequence is in the .dat file where the first line contains
the number of residues and each consecutive line contains a residue name.

### Files

| option | filename | type | description |
| --- | --- | --- | --- |
| **-f** | nnnice.dat | Input | Generic data file |
| **-o** | plot.eps | Output | Encapsulated PostScript (tm) file |

### Other options

| option | type | default | description |
| --- | --- | --- | --- |
| **-[no]h** | bool | no | Print help info and quit || **-[no]version** | bool | no | Print version info and quit || **-nice** | int | 19 | Set the nicelevel || **-r0** | int | 1 | The first residue number in the sequence || **-rot0** | real | 0 | Rotate around an angle initially (90 degrees makes sense) || **-T** | string |  | Plot a title in the center of the wheel (must be shorter than 10 characters, or it will overwrite the wheel) || **-[no]nn** | bool | yes | Toggle numbers |

---

http://www.gromacs.org  
gromacs@gromacs.org
